# Turning trash into treasure: *Hermetia illucens* microbiome and biodegradation of industrial side streams

**DOI:** 10.1128/aem.00991-24

**Published:** 2024-10-22

**Authors:** Patrick Klüber, Friscasari F. Gurusinga, Sabine Hurka, Andreas Vilcinskas, Dorothee Tegtmeier

**Affiliations:** 1 Branch for Bioresources, Fraunhofer Institute for Molecular Biology and Applied Ecology (IME), Giessen, Germany; 2 BMBF Junior Research Group in Bioeconomy (BioKreativ) “SymBioÖkonomie”, Giessen, Germany; 3 LOEWE Centre for Translational Biodiversity Genomics (LOEWE-TBG), Frankfurt, Germany; 4 Institute for Insect Biotechnology, Justus Liebig University, Giessen, Germany; Norwegian University of Life Sciences, Ås, Norway

**Keywords:** insect rearing, fiber, cellulolytic bacteria, sustainability, insect development, life-history traits

## Abstract

Black soldier fly larvae (BSFL) have attracted attention due to their ability to upcycle various biological side streams into valuable biomass, such as proteins, lipids, and chitin. In this study, we investigated the impact of high-fiber diets on larval growth performance and the shift of microbes in the gut. We tested empty fruit bunches (EFB), potato pulp (PP), and cottonseed press cake (CPC), with chicken feed (CF) used as a control diet. We found that larvae reared on the EFB, PP, and CPC were smaller than control larvae at the end of development due to the low nutritional value of the diets. However, survival rates of more than 90% were observed regardless of the diet. We used a cultivation-dependent approach to analyze the microbial community in the gut of BSFL, isolated, and identified a total of 329 bacterial strains. *Bacillaceae* were most frequently isolated from larvae reared on the high-fiber EFB diet. These isolates were predicted to degrade cellulose *in silico* and this was subsequently confirmed *in vitro* using the Congo Red assay. Whereas the members of *Enterobacteriaceae* and *Morganellaceae* were mostly found in guts of larvae reared on the high-protein diets CPC and CF. We conclude that the gut microbiome plays a crucial role in the digestion of fiber-rich plant organic material, thereby enabling the BSFL to successfully complete their life cycle also on substrates with low nutritional value. As a result, BSFL convert industrial side streams into valuable biomass, reducing waste and promoting sustainability.

**IMPORTANCE:**

Organic side streams from various industries pose a challenge to the environment. They are often present in huge amounts and are mostly discarded, incinerated, used for biogas production, or as feed for ruminant animals. Many plant-based side streams contain difficult-to-digest fiber as well as anti-nutritional or even insecticidal compounds that could harm the animals. These challenges can be addressed using black soldier fly larvae, which are known to degrade various organic substrates and convert them into valuable biomass. This will help mitigate agro-industrial side streams via efficient waste management and will contribute to the more economical and sustainable farming of insects.

## INTRODUCTION

Insects play an increasingly important role as an alternative source of protein for the sustainable production of food and feed. Larvae of the black soldier fly (*Hermetia illucens*) have an unusually low dietary specialization and are regarded as generalists. Therefore, they can break down a variety of substrates derived from organic waste and industrial side streams and convert them into valuable proteins, lipids, and chitin. This ability, combined with their high rate of reproduction, fast growth, and rapid life cycle, makes them one of the most economically important farmed insects in the world (1, 2).

Similar to many other animals, *H. illucens* relies on a beneficial gut microbiome that includes bacteria, fungi, and archaea (3, 4). The insect-associated microbes are involved in critical processes including (i) development of the gastrointestinal tract, (ii) modulation of the immune system, (iii) digestion of organic matter and detoxification of compounds, (iv) the provision of essential amino acids and vitamins, and (v) the synthesis of pheromones and kairomones required for communication (3, 5–9). Moreover, beneficial microbes occupy niches in the host’s gut to prevent colonization by pathogens and parasites through direct competition or by stimulating the synthesis of antimicrobial peptides (AMPs) (4, 7, 10, 11).

Black soldier fly larvae (BSFL) are known to grow even on substrates with a high microbial load (e.g., feces), suggesting a highly adaptive and dynamic immune response. They can even change the microbial composition of the substrate (12, 13) and reduce the number of pathogens such as *Salmonella* spp. and enterohemorrhagic *Escherichia coli* O157:H7 in manure and aquaculture waste (14–16).

To date, most studies on BSFL microbes have been culture-independent and based on next-generation sequencing. But, to fully realize their biotechnological potential, current studies still rely on culture-dependent approaches (9, 17, 18). Nevertheless, functional parallels were drawn between the gut microbiome of insects and conventional farm animals, indicating a significant contribution to productivity and health (3). The utilization of versatile and often recalcitrant feed substrates by BSFL is strongly dependent on their microbiome (19–22). In particular, BSFL are not naturally specialized to break down fiber-rich substrates and do not have the physiological adaptations necessary for this purpose such as mechanical breakdown, gut pH milieu, and endogenous enzymes (23). Accordingly, the degradation of robust plant biopolymers such as cellulose and lignin would not be possible without the enzyme repertoire of associated gut microbes. Bacteria possess multiple carbohydrate-active enzymes (CAZymes), namely endoglucanases, exoglucanases, and β-glucosidases, which hydrolyze cellulose (24). Such beneficial insect–microbe interactions have already been described in representatives of the orders Coleoptera, Hymenoptera, Isoptera, and Orthoptera (5, 25–27).

BSFL feed on a variety of substrates, and the microbial composition and diversity of their gut therefore shifts dynamically to reflect the type of nutrients in the feed (8, 19, 22, 28–31). By contrast, the termite microbiome is determined by the host due to dietary restrictions (e.g., wood-feeding, soil-feeding, or fungus-cultivating termites) (26, 27). The diet-dependent expression of more than 50 putative AMPs also plays a pivotal role in the regulation of the gut microbiome (7), although an omnipresent core community is thought to exist (20, 32, 33). Representatives of the genera *Enterococcus*, *Morganella*, *Providencia*, *Klebsiella,* and *Actinomyces* can be considered as an integral part of the bacterial core gut microbiome of BSFL (8, 9, 12, 13, 20, 21, 31–38). The inoculation of the substrate with companion bacteria such as *Bacillus subtilis* or *Bacillus licheniformis* had positive effects on the growth rate, developmental time, and final larval weight of BSFL (17, 39). Feeding status also influences the microbiome and related metabolic capabilities, potentially reducing larval growth performance and feed conversion efficiency (40). Homeostasis of the BSFL gut microbiome therefore regulates feed degradation capacity and insect health; otherwise, the larvae would be less adaptable and more restricted in terms of their feed spectrum.

Industrial side streams play an important role as feed sources to ensure the economic competitiveness of farmed insects. Cottonseed press cake (CPC), empty fruit bunches (EFB) from the palm oil industry, and potato pulp (PP), a byproduct of starch manufacture, are currently used inefficiently, partly because of their low nutritional value. For example, EFB contains large amounts of fiber but only low levels of protein and lipids (41). By contrast, CPC is rich in proteins and lipids but is unsuitable as feed for several animals due to the presence of the anti-nutritional and insecticidal substance gossypol (42–45). Nevertheless, it can be used as the sole feed for BSF rearing (32). In this study, we analyzed the development and physiological performance of the BSF reared on CPC, EFB, or PP and analyzed their gut microbial communities, in comparison to data from previous studies in which BSFL were reared on a standard chicken feed (CF) diet (9, 32). We used a cultivation-dependent approach to assess viable, metabolically active gut bacteria and to test the resulting pure cultures for their ability to break down cellulose *in vitro*. Finally, we predicted the metabolic functions of 486 isolates from BSFL guts using bioinformatic tools and linked them to diet-dependent shifts.

## RESULTS

### Development and life-history traits

**Fig 1:**
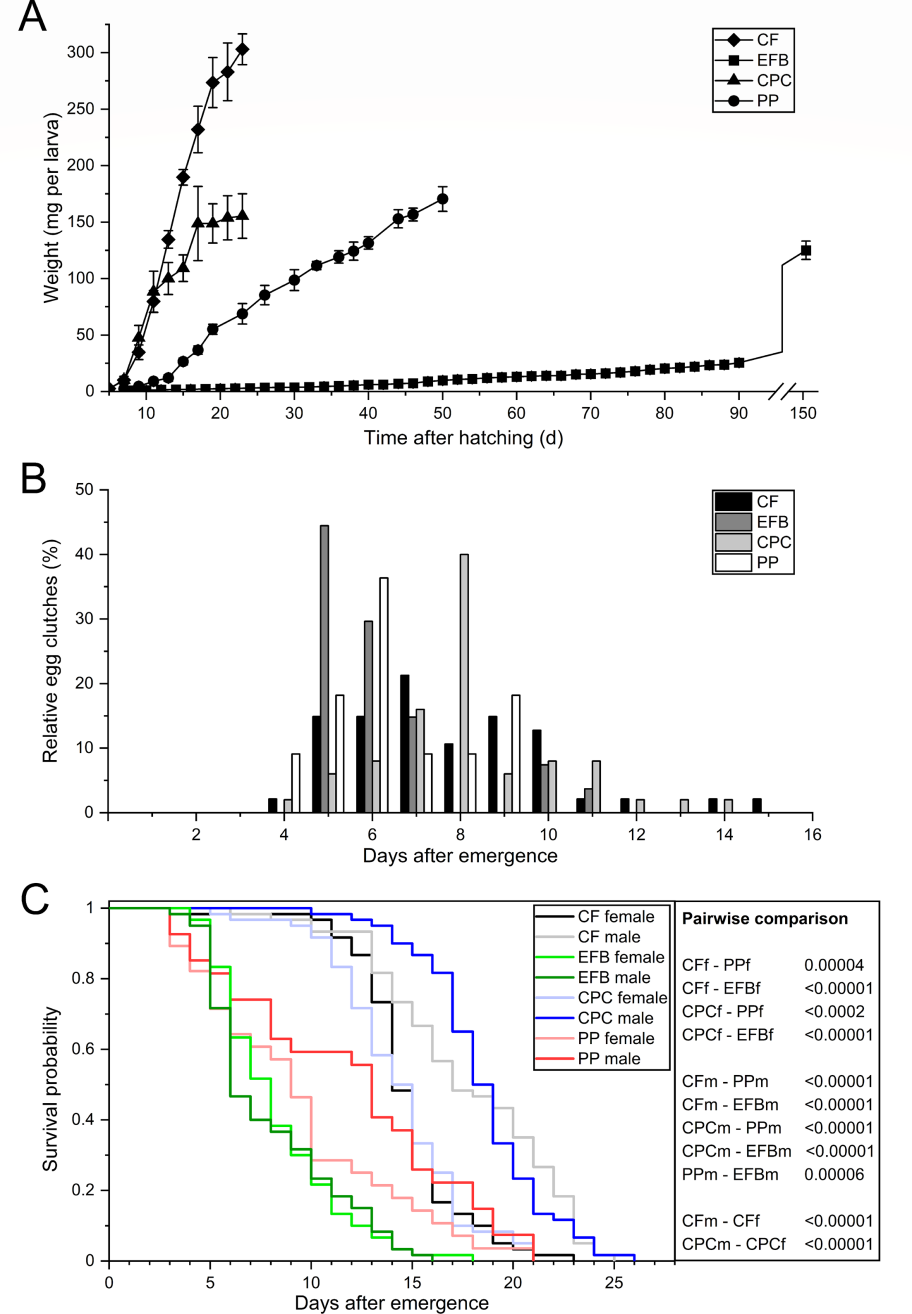
Growth curves (**A**) and temporal course of oviposition (**B**) of BSF fed with CF, EFB, CPC, and PP. (**C**) Sex-dependent Kaplan-Meier survival functions of 30 pairs per diet. *P*-values represent diets compared pairwise via log-rank test (f = female; m = male).

We evaluated the growth and life-history traits of BSF raised on agro-industrial side streams (EFB, CPC, and PP) compared to a standard diet (CF). The time until ≥50% of the eggs hatched ranged from 3.0 to 4.0 d (Table 1) and did not differ significantly across dietary groups (*F*_(3, 8)_ =1.14; *P* = 0.39). All diets affected larval development (*Welch’s F*_(3, 7.47)_ =26748.84; *P* < 0.00001). Larvae reared on CF and CPC had the shortest developmental periods, followed by PP, with a ≥2.1-fold increase (*M* = 27.67; *SE* = 0.33; *P* = 0.0004 and *M* = 26.35; *SE* = 0.38; *P* = 0.0005). However, EFB extended larval development significantly to 151.3 d (*M* = 101.33; *SE* = 0.33; *P* = 0.0003; Fig. 1A). There was no significant difference in prepupal development between groups (*F*_(3, 8)_ =3.90; *P* > 0.05), whereas intrapuparial metamorphosis was accelerated by 1.6-fold on the CPC diet and 2-fold on the PP diet compared to CF (*F*_(3, 8)_ =8.89; *P* ≤ 0.006). The total duration of development was the shortest in the CF and CPC groups (*Welch’s F*_(3, 4.06)_ =21092.07; *P* < 0.00001). PP and EFB caused significant ≥1.5-fold (*M* = 20.64; *SE* = 1.94; *P* = 0.002 and *M* = 24.65; *SE* = 1.55; *P* = 0.008) and ≥3.6-fold increases in the total development time, respectively (*M* = 121.33; *SE* = 1.25; *P* = 0.00009 and *M* = 125.35; *SE* = 0.44; *P* < 0.00001), compared with the aforementioned treatments. Larva-to-prepupa developmental success was consistently high at 99.3%–100% in all feeding groups (*Welch’s F*_(3, 20.00)_ =0.00; *P* = 1.00). Conversely, prepupa-to-pupa developmental success was ≥7.6% higher for larvae reared on the different side streams compared to CF (*Welch’s F*_(3, 12.80)_ =19.96; *P* = 0.00004). The proportion of adult flies emerging from pupae did not differ significantly between the side streams and CF, but the developmental success of pupae reared on PP was lower than that of their conspecifics grown on CPC and EFB (*F*_(3, 8)_ =5.95; *P* ≤ 0.007).

**TABLE 1:** Temporal, physiological, and reproductive parameters of BSF fed with CF, EFB, CPC, and PP^*a*^

Parameter		Sampling size	CF^*b*^	EFB	CPC^*b*^	PP
Hatching time (d)		≥ 50%	3.0 ± 0.8^a^	3.3 ± 0.5^a^	3.0 ± 0.8^a^	4.0 ± 0.0^a^
Larval development (d)^*c*^		≥ 50%	22.3 ± 0.5^a^	151.3 ± 0.5^b^	23.7 ± 0.5^a^	50.0 ± 0.0^c^
Prepupa-pupa (d)		*n* = 300	11.3 ± 1.7^a^	6.0 ± 2.2^a^	10.0 ± 0.0^a^	10.7 ± 2.1^a^
Intrapuparial metamorphosis (d)^*d*^		*n* = 300	10.7 ± 0.5^a^	8.0 ± 1.6^ab^	6.7 ± 0.5^b^	5.3 ± 1.2^b^
Adult preoviposition period (d)^*e*^		*n* = 30	7.7 ± 0.8^a^	6.2 ± 0.1^ab^	8.2 ± 0.6^ac^	6.4 ± 0.3^a^
Total preoviposition period (d)^*f*^		*n* = 30	52.0 ± 2.0^a^	171.5 ± 0.9^b^	48.5 ± 1.1^a^	72.4 ± 2.2^c^
Total development (d)^*g*^		*n* = 300	47.3 ± 1.7^a^	168.7 ± 0.5^b^	43.4 ± 1.8^a^	70.0 ± 2.2^c^
Oviposition period (d)		*n* = 30	7.3 ± 2.1^a^	5.3 ± 0.5^a^	6.7 ± 0.5^a^	3.3 ± 1.7^a^
Oviposition peak (d)		*n* = 30	7.6 ± 1.0^a^	5.3 ± 0.5^ab^	7.5 ± 0.4^ac^	6.0 ± 0.0^a^
Oviposition span (min-max d)		*n* = 30	4–15	5–11	4–14	4–9
Successful development larva-prepupa (%)		*n* = 300	100.0 ± 0.0^a^	99.3 ± 0.9^a^	99.3 ± 0.9^a^	100.0 ± 0.0^a^
Successful development prepupa-pupa (%)		*n* = 300	90.7 ± 0.5^a^	98.3 ± 1.3^b^	100.0 ± 0.0^b^	100.0 ± 0.0^b^
Successful development pupa-adult (%)		*n* = 300	97.0 ± 2.4^a^	99.3 ± 0.9^ab^	99.7 ± 0.5^ab^	92.7 ± 2.6^ac^
Adult longevity (d)	♂	*n* = 30	17.7 ± 0.5^a^	7.9 ± 1.3^b^	18.5 ± 0.5^a^	11.7 ± 1.9^c^
	♀	*n* = 30	14.7 ± 0.2^a^	8.2 ± 0.8^b^	14.3 ± 1.4^a^	9.3 ± 1.1^b^
	total	*n* = 60	16.2 ± 0.2^a^	8.1 ± 1.0^b^	16.4 ± 0.9^a^	10.5 ± 1.5^c^
Adult longevity (min-max d)	♂	*n* = 30	6–25	3–16	10–26	3–21
	♀	*n* = 30	4–23	4–18	5–21	3–21
	total	*n* = 60	4–25	3–18	5–26	3–21
Final larval weight (mg)		*n* = 150	303.0 ± 13.6^a^	125.1 ± 8.1^b^	155.3 ± 16.1^bc^	170.5 ± 10.9^c^
Final larval length (mm)		*n* = 150	26.0 ± 0.1^a^	19.8 ± 0.1^b^	18.7 ± 0.2^c^	15.0 ± 0.1^d^
Weight prepupa (mg)		*n* = 150	219.6 ± 18.7^a^	88.3 ± 2.8^b^	101.8 ± 9.6^b^	187.3 ± 25.3^a^
Length prepupa (mm)		*n* = 150	23.4 ± 0.1^a^	16.2 ± 0.2^b^	16.8 ± 0.4^c^	19.3 ± 0.4^d^
Weight pupa (mg)		*n* = 150	169.0 ± 10.7^a^	72.0 ± 2.5^b^	93.9 ± 4.9^c^	140.2 ± 12.6^d^
Length pupa (mm)		*n* = 150	22.7 ± 0.1^a^	16.1 ± 0.2^b^	17.0 ± 0.2^c^	18.5 ± 0.6^d^
Weight adult (mg)	♂	*n* = 90	89.4 ± 8.5^a^	24.4 ± 4.1^b^	40.2 ± 4.0^c^	43.4 ± 2.6^c^
	♀	*n* = 90	103.6 ± 8.3^a^	36.4 ± 4.6^b^	59.1 ± 3.6^c^	55.6 ± 3.6^c^
	total	*n* = 180	98.1 ± 8.0^a^	31.1 ± 3.4^b^	50.6 ± 3.5^c^	49.8 ± 3.4^c^
Length adult (mm)^*h*^	♂	*n* = 90	16.8 ± 0.3^a^	12.4 ± 0.4^b^	14.4 ± 0.2^c^	11.6 ± 0.2^d^
	♀	*n* = 90	17.5 ± 0.3^a^	13.4 ± 0.1^b^	15.8 ± 0.2^c^	12.0 ± 0.3^d^
	total	*n* = 180	17.2 ± 0.2^a^	12.9 ± 0.3^b^	15.2 ± 0.2^c^	11.8 ± 0.3^d^
Sex ratio (♀/♂)		*n* = 180	1.5 ± 0.2^a^	1.0 ± 0.2^a^	1.2 ± 0.1^a^	1.3 ± 0.6^a^
Fecundity (egg clutches/10 females)		*n* = 30	7.8 ± 0.9^a^	4.5 ± 0.7^b^	8.3 ± 0.8^a^	3.9 ± 0.8^b^
Egg clutch size (eggs/clutch)		*n* = 10	676.0 ± 59.6^a^	452.8 ± 99.3^b^	629.9 ± 65.4^a^	804.0 ± 271.1^a^
Span of egg clutch size (min-max eggs)		*n* = 10	548–763	243–551	530–729	557–1494
Egg clutch weight (mg)		*n* = 10	16.5 ± 3.8^a^	10.2 ± 2.7^b^	12.4 ± 2.0^ab^	19.7 ± 7.2^a^
Egg weight (mg/ egg)^*i*^		*n* = 10	0.024 ± 0.005^a^	0.022 ± 0.002^ab^	0.020 ± 0.002^ac^	0.024 ± 0.003^ab^

^*a*^ Data are means ± SD. Statistically significant differences (*P* < 0.05; one-way ANOVA or Welch’s ANOVA; Kaplan-Meier estimation for data related to longevity) between diets are indicated by different letters (a–d).

^*b*^ Data for CPC (except for fecundity and longevity-related parameters) and CF have been published before (23, 32) and are included here for comparison.

^*c*^ Period from hatching to ≥50% prepupae.

^*d*^ Period from pupation to ≥50% adults emerged.

^*e*^ Period from adult emerging to first oviposition.

^*f*^ Period from hatching to first oviposition.

^*g*^ Period from oviposition to adults emerging.

^*h*^ Defined as distance between antennal attachments and abdominal genitalia.

^*i*^ Calculated by dividing the clutch weight by the egg count.

We observed no significant effect on the adult preoviposition period when comparing the side stream groups and CF counterparts (*F*_(3, 8)_ =6.56; *P* ≤ 0.43). By contrast, the adult preoviposition period was 2 days shorter in the EFB group compared with CPC (*F*_(3, 8)_ =6.56; *P* = 0.006). The total preoviposition period of the CF and CPC groups was the same but differed significantly from the other treatments (*F*_(3, 8)_ =1693.16; *P* < 0.00001). Generally, individuals reared on EFB took 2.4-fold to 3.3-fold longer to deposit eggs than the other groups (*F*_(3, 8)_ =1693.16; *P* < 0.00001). There were no significant differences in the oviposition period of pairs reared on different side streams or CF (*F*_(3, 8)_ =3.29; *P* = 0.08), but the mean values ranged from 3.3 to 7.3 d. In BSF that laid eggs, oviposition peaked at 5.3–7.6 d post-emergence (*Welch’s F*_(3, 6.99)_ =7.09; *P* < 0.02) and was the same in the side streams and CF, but differed significantly between pairs fed on CPC and EFB (*M* = −2.13; *SE* = 0.44; *P* < 0.03). CF led to the longest oviposition span of 12 d (days 4–15), followed by CPC with 11 d (days 4–14), EFB with 7 d (days 5–11) and PP with 6 days (days 4–9) (Fig. 1B; Table 1). Females in the EFB group laid 88.9% of clutches within a 3-day period (days 5–7), while those in the CF or PP groups laid 51.1% and 63.6% of clutches in the same interval, respectively. CPC females laid 64.0% of clutches with a 1-day offset (days 6–8). Interestingly, females in the CPC and EFB groups deposited a large proportion of clutches (40.0%–44.4%) within only 24 h (day 8 and day 5). In general, ≥78% of egg clutches from all treatments were found within 9 d post-emergence, while only scattered egg clutches were deposited at the end of the oviposition period, accounting for a relative proportion of ~2% (Fig. 1B).

Total adult longevity in the CF and CPC groups was the same but differed significantly from the other treatments (*χ^2^* = 278.45; *P* < 0.00001). The same pattern was also observed for male (*χ^2^* = 22.36; *P* < 0.00001) and female longevity (*χ^2^* = 14.35; *P* < 0.0002). In general, CF and CPC were associated with the greatest sex-dependent longevity in adults (Fig. 1C; Table 1). Accordingly, EFB and PP led to a significant 1.8-fold to 2.2-fold (*χ2* = 80.83; *P* < 0.00001) or 1.5-fold to 1.6-fold reduction (*χ2* = 16.74; *P* = 0.00004) compared to CF, respectively. Males and females in the EFB (*χ^2^* = 0.13; *P* = 0.72) and PP groups (*χ^2^* = 2.85; *P* = 0.09) had similar lifespans. EFB flies had the shortest lifespans in both sexes at 7.9–8.2 d compared to any other diet (*χ^2^* = 17.14; *P* ≤ 0.00003). By contrast, CF and CPC males lived 3.0 d (*χ^2^* = 24.54; *P* < 0.00001) and 4.2 d longer (*χ^2^* = 41.13; *P* < 0.00001), respectively, than the corresponding females. Individual male longevity ranged from 3 d (EFB and PP) to 26 d (CPC). On the other hand, females lived for at least 3 d (PP) and at most 23 d (CF).

All diets allowed larvae to complete their entire life cycle, including mating and oviposition, but none of the larvae on the agro-industrial side streams exceeded the performance of the CF group. Final larval weight (*F*_(3, 8)_ =84.12; *P* < 0.00001) and length (*Welch’s F*_(3, 329.56)_ =2517.17; *P* < 0.00001) differed significantly between the groups, with the heaviest and largest larvae reared on CF, followed by those reared on PP (Fig. 1A; Table 1). We also observed the same pattern for weight (*F*_(3, 8)_ =30.24; *P* = 0.0001 and *F*_(3, 8)_ =50.51; *P* = 0.00002) and length (*Welch’s F*_(3, 353.10)_ =1215.51; *P* < 0.00001 and *Welch’s F*_(3, 347.55)_ =1308.49; *P* < 0.00001) at the subsequent prepupal and pupal stages. Here, larvae reared on EFB showed the lowest larval, prepupal, and pupal weights, with values 1.2-fold to 2.1-fold lower than the CPC and PP groups. Larvae fed on CF produced adult males and females that were both significantly heavier (*F*_(3, 8)_ =55.91; *P* = 0.00001 and *F*_(3, 8)_ =55.67; *P* = 0.00001) and longer (*Welch’s F*_(3, 278.31)_ =876.04; *P* < 0.00001 and *Welch’s F*_(3, 333.75)_ =1199.68; *P* < 0.00001) than their counterparts on side streams. EFB males and females were the lightest (*F*_(3, 8)_ =65.73; *P* < 0.00001), followed by those in the CPC and PP groups, which showed no significant differences in weight compared to each other (*F*_(3, 8)_ =65.73; *P* = 0.86). Regardless of sex, adults in the PP group were the shortest (*Welch’s F*_(3, 616.93)_ =1820.06; *P* < 0.00001), but those in the CPC and EFB groups were up to 31.6% and 11.7% longer, respectively. In general, females were 15.9%–49.2% heavier and 3.4%–9.7% longer than the corresponding males, regardless of the diet. The sex ratio did not differ significantly between the groups (*F*_(3, 8)_ =0.71; *P* = 0.57). The fecundity of CF and CPC flies was the same but was significantly (1.7-fold to 2.1-fold) lower in the other groups (*F*_(3, 8)_ =16.07; *P* < 0.001). Furthermore, the egg clutch size in the EFB group was 1.4-fold to 1.8-fold lower (*Welch’s F*_(3, 19.04)_ =11.72; *P* = 0.0001) than in all other groups, and the egg clutch weight was 1.6-fold to 1.9-fold lower than in the CF and PP groups (*Welch’s F*_(3, 18.86)_ =7.86; *P* = 0.001). The individual egg clutches consisted of a minimum of 243 (EFB) and a maximum of 1494 (PP) eggs. No significant effect on single egg weight was found between the side stream groups and their CF counterparts (*Welch’s F*_(3, 19.13)_ =7.00; *P* = 0.002). Interestingly, a second oviposition event occurred in four PP pairs 1–6 d after the initial oviposition, whereas multiple ovipositions were not observed in any other group.

### Nutritional parameters of feed substrates and BSFL

The dry matter content differed significantly between the diets, with PP containing up to 3.8-fold more water than the others (*Welch’s F*_(3, 4.12)_ =2472.24; *P* < 0.00001; Table 2). CF contained more than twice as much crude ash as the agro-industrial side streams (*Welch’s F*_(3, 3.48)_ =48.38; *P* = 0.005). The crude fiber content was highest in EFB and CPC and differed significantly by more than 35% from CF and PP (*F*_(3, 8)_ =1868.18; *P* < 0.00001). The EFB and CPC diets had the greatest crude fat content, differing significantly from CF and PP, the latter containing only 0.2% DM of fat (*Welch’s F*_(3, 3.89)_ =475.33; *P* = 0.00001). Total nitrogen (*F*_(3, 8)_ =1491.73; *P* < 0.00001) and crude protein calculated from it (*Welch’s F*_(3, 4.77)_ =1039.51; *P* < 0.00001) differed significantly across the diets. CPC was the most protein-rich, followed by CF with 8.0% less protein.

**TABLE 2:** Chemical composition of the diets CF, EFB, CPC and PP, and the corresponding larvae reared on them^*a*^

	CF	EFB	CPC	PP
Feed				
Dry matter (%)	91.4 ± 0.1^a^	91.9 ± 0.1^b^	92.5 ± 0.1^c^	24.2 ± 1.0^d^
Crude ash (%DM)	12.9 ± 0.9^a^	5.5 ± 0.3^b^	4.8 ± 0.1^bc^	5.9 ± 0.3^bd^
Crude fiber (%DM)	9.9 ± 2.7^a^	60.3 ± 6.7^b^	45.7 ± 3.5^c^	10.8 ± 3.4^a^
Crude fat (%DM)	2.5 ± 0.1^a,*b*^	7.6 ± 0.2^b^	8.7 ± 0.5^b,*b*^	0.2 ± 0.2^c^
Total nitrogen (%DM)	2.9 ± 0.1^a^	0.9 ± 0.1^b^	4.2 ± 0.1^c^	1.1 ± 0.0^d^
Crude protein (%DM)	18.2 ± 0.4^a^	5.9 ± 0.2^b^	26.2 ± 0.7^c^	7.0 ± 0.1^d^
Larvae				
Dry matter (%)	35.7 ± 0.1^a^	28.5 ± 0.2^b^	33.0 ± 0.6^a^	25.9 ± 1.1^b^
Crude ash (%DM)	4.9 ± 0.2^a^	8.4 ± 0.2^b^	4.7 ± 0.3^a^	5.9 ± 0.3^c^
Crude fat (%DM)	33.9 ± 1.1^a,*b*^	26.0 ± 0.7^b^	23.3 ± 1.2^c,*b*^	16.8 ± 0.7^d^
Total nitrogen (%DM)	6.5 ± 0.2^a^	6.2 ± 0.5^ab^	8.0 ± 0.1^b^	7.9 ± 0.1^b^
Crude protein (%DM)	40.7 ± 1.3^a^	38.4 ± 3.3^ab^	50.0 ± 0.1^b^	49.6 ± 0.3^b^

^*a*^ Besides the dry matter content, values are given as a percentage of dry mass (%DM). Data are means ± SD. Statistically significant differences (*P* < 0.05; one-way ANOVA or Welch’s ANOVA) between diets are indicated by different letters (a–d).

^*b*^ Data for the crude fat content of CF and CPC (feed and larvae) have been published before (32) and are included here for comparison.

CF and CPC resulted in a significantly higher larval dry matter content than EFB and PP (*Welch’s F*_(3, 3.40)_ =1140.69; *P* = 0.00004; Table 2). However, the crude ash content of larvae reared on CF and CPC was significantly lower than that of larvae on the other diets (*F*_(3, 9)_ =114.87; *P* < 0.00001). All diets affected the larval crude fat content, and larvae in the CF group were able to build up the most fat, even though the fat content of the diet was only 2.5% DM (*F*_(3, 8)_ =110.94; *P* < 0.00001). Concomitantly, despite the very low crude fat content of the diet, PP larvae accumulated an 84-fold higher amount of fat in their biomass. Total nitrogen and crude protein (*Welch’s F*_(3, 4.36)_ =70.43; *P* = 0.0006) levels were significantly (1.2-fold) higher in larvae reared on CPC and PP compared with CF.

We also clarified the extent to which the dietary nutritional parameters of crude fat, protein, and fiber were linearly related to BSF life-history traits. Critical temporal aspects, including larval development time (*r* = −0.72; *P* = 0.008), total preoviposition period (*r* = −0.71; *P* = 0.009), and total development time (*r* = −0.72; *P* = 0.008), were negatively correlated with increasing crude protein, indicating that higher protein levels significantly shorten the time until harvest and the generation time in general. Both female (*r* = 0.60; *P* < 0.001) and male longevity (*r* = 0.71; *P* < 0.001) were positively correlated with increasing crude protein levels. Moreover, oviposition peaked later (*r* = 0.77; *P* = 0.003) and the fecundity improved with increasing crude protein levels (*r* = 0.89; *P* = 0.0001). As opposed to the protein content, fiber content was positively correlated with larval development time (*r* = 0.68; *P* < 0.02), total preoviposition period (*r* = 0.67; *P* < 0.02), and total development time (*r* = 0.66; *P* = 0.02), indicating that higher fiber levels significantly extend the time until harvest and the generation time. Both female (*r* = −0.32; *P* < 0.00001) and male longevity (*r* = −0.38; *P* < 0.00001) were negatively correlated with increasing fiber levels, while fecundity and oviposition peak were not linearly related to dietary fiber content. The egg clutch weight was negatively correlated with increasing fiber levels (*r* = −0.60; *P* = 0.00004). By contrast, none of these life-history traits was linearly related to the dietary crude fat content, but egg clutch weight was negatively correlated with increasing fat levels (*r* = −0.60; *P* = 0.00004).

### Cultivation, isolation, and identification of gut bacteria

We isolated a total of 329 bacterial strains from the BSFL gut samples, 119 from larvae reared on CPC (07-batch), 98 from those reared on EFB (06-batch), and 112 reared on PP (10-batch). We obtained partial 16S rRNA sequences (forward and/or backward reads) from all isolates, ranging from 117 to 1,207 bp. Only sequences with a length >275 bp were used for further analysis. We gained five pairs of identical forward-read sequences. As we did not have any backward reads to differentiate them, we handled each pair as one, as follows: 06–016f + 06–029f, 06–050f + 06–059f, 06–065f + 06–087f, 07–015f + 07–019f, and 07–101f + 07–125f. This leads to 117 and 95 unique isolates from CPC and EFB rearing, respectively. For comparison, we included 162 isolates obtained from larvae reared on CF (9).

**Fig 2:**
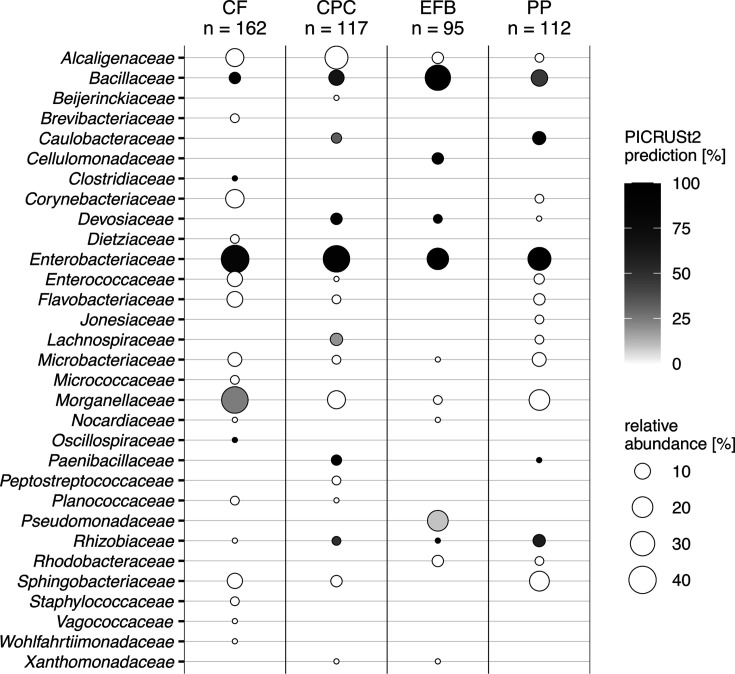
Bubble chart showing the family-level composition of the bacterial isolates based on 16S rRNA sequence. Gut bacterial isolates from BSFL reared on CF, EFB, CPC, and PP diets are shown in 31 classified families. Families without a suitable classification (uncultivated, undefined, and not applicable) are excluded. The relative abundance (%) is shown in different bubble sizes, while a color gradient that runs from white to black indicates the number of isolates that are predicted to have genes for endoglucanases involved in cellulose degradation (%).

We were able to cultivate representatives from 44 different genera in total. In all, 24 different genera were obtained from the CPC group, 16 from the EFB group, and 27 from the PP group (Table S3). Most gut isolates from the CF, CPC, and PP groups represented the family *Enterobacteriaceae* (25.3%, 31.6%, and 23.2%, respectively) and *Morganellaceae* (22.8%, 12.0%, and 17.9%, respectively), followed by *Corynebacteriaceae* (9.3%) in the CF group, *Alcaligenaceae* (22.2%) in the CPC group, and *Sphingobacteriaceae* (16.1%) in the PP group. *Bacillaceae* were the most frequently isolated (34.7%) bacteria in the EFB group, followed by *Pseudomonadaceae* (21.1%). We also isolated members of the *Enterococcaceae* from the CF (5.6%), PP (2.7%), and CPC diet groups (0.9%). Members of the *Cellulomonadaceae* (4.2%) were only obtained from the EFB diet group (Fig. 2; Table S2).

*Alcaligenes* (18.8%), *Klebsiella* (17.1%), *Citrobacter* (14.5%), and *Morganella* (10.3%) were the most abundant genera among the CPC isolates. These genera were also frequently isolated from the CF diet group, especially *Klebsiella* (16.7%) and *Morganella* (13.0%), which were the most frequent isolates. Furthermore, *Corynebacterium* (9.3%) was frequently isolated from the CF diet group. *Sphingobacterium* (15.2%), *Morganella* (15.2%), *Citrobacter* (12.5%), and *Bacillus* (8.9%) were the most abundant isolates in the PP group. *Bacillus* was the most abundant genus (34.7%) in the EFB group, followed by *Pseudomonas* (21.1%) and *Citrobacter* (14.7%) (Table S3). By contrast, the genera *Morganella* and *Alcaligenes* were absent or rarely isolated from the EFB diet group, whereas *Pseudomonas* was not isolated from the other diet groups. The detailed results of the classifications based on BLAST queries against the NCBI nucleotide database are shown in Table S1.

### Prediction of microbial functions

**Fig 3:**
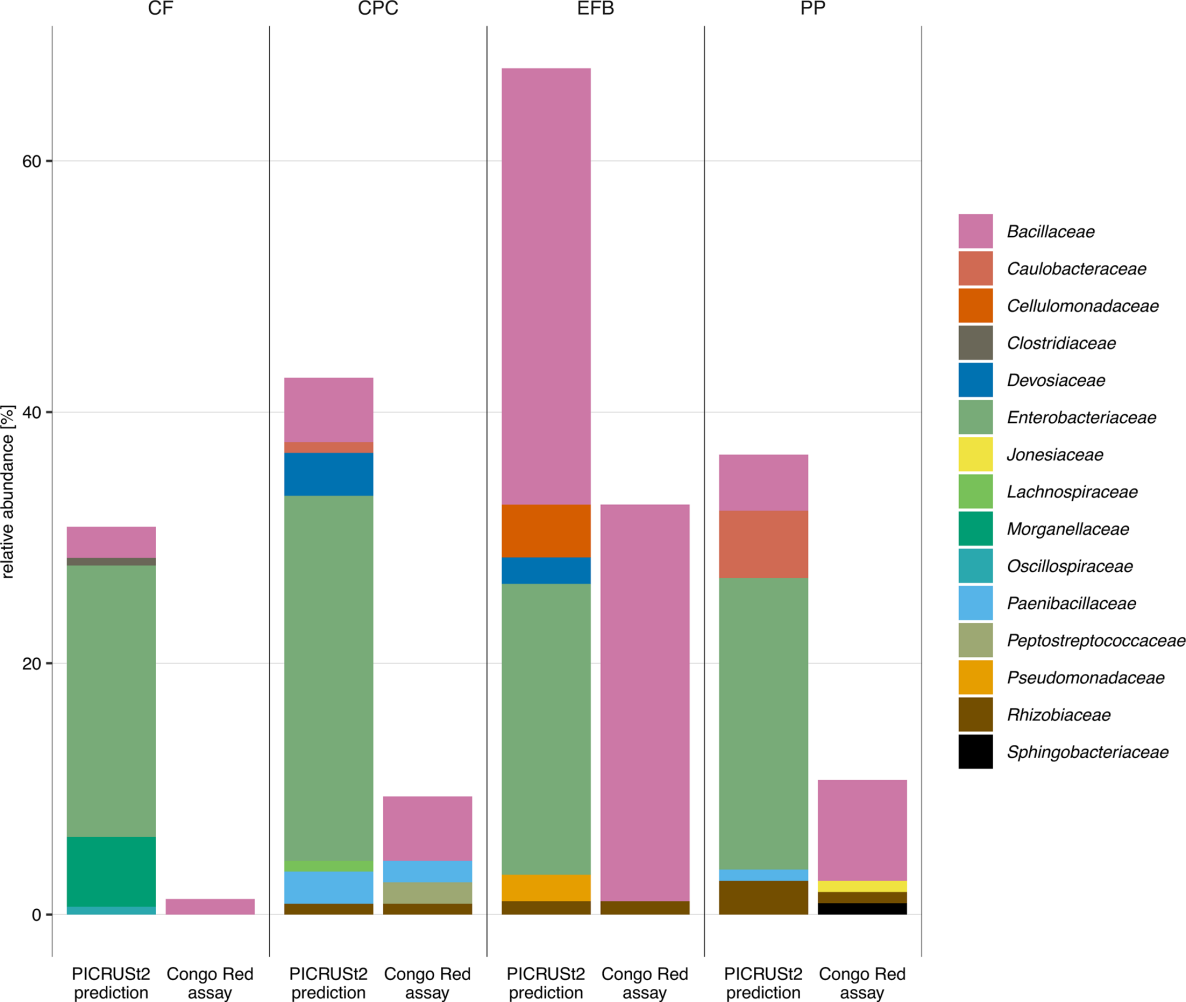
Comparison of *in silic*o (PICRUSt2) and *in vitro* (Congo Red) analysis of the gut bacterial isolates from BSFL reared on CF, EFB, CPC, and PP at the family level. The bar chart describes the relative abundance of isolates that were predicted to have genes for endoglucanases involved in cellulose degradation, whereas *in vitro* analysis shows the relative abundance of positive isolates with Congo Red assay.

We used PICRUSt2 to identify potential cellulose- and lignin-degrading bacteria based on the 16S rRNA sequence. Here, we obtained the number of isolates which were predicted to degrade cellulose. We found 64 isolates (67.4%) from the EFB group, 50 isolates (42.7%) from the CPC group, followed by the PP group which results in 41 isolates (36.6%) and 50 isolates (30.9%) from the CF group. The prediction showed that the larvae reared on CPC and EFB were enriched in potential cellulose-degrading bacteria when compared to the control group (CF) (Fig. 3). *Bacillaceae* and *Enterobacteriaceae* were the most prominent families within our strain collection predicted to degrade cellulose. Despite the prediction of cellulolytic activity, members of several families including *Clostridiaceace, Cellulomonadaceae, Devosiaceae, Lachnospiraceae*, and *Pseudomonadaceae* were negative in Congo Red assay (Fig. 3). Furthermore, no prediction was found for the genes for exoglucanases using PICRUSt2. Several enzymes are involved in lignin degradation, such as laccase, lignin peroxidase, manganese peroxidase, and versatile peroxidase. We only found the modules for laccase, but no predicted genes were found in our data using PICRUSt2.

### Evaluation of cellulose degradation

To confirm the function prediction, we tested the strains for their cellulose-degradation capacity *in vitro* via Congo Red assay. *Cellulomonas flavigena* (DSM20109) has enzymes that play a role in microbial cellulose degradation (46) and was used as the positive control in this experiment. Here, we identified 56 strains (CF: 2 strains, EFB: 31 strains, CPC: 11 strains, PP: 12 strains) that formed a clear zone indicating their ability to hydrolyze cellulose (Table 3). The activity was quantified by calculating the enzymatic activity index, which varied from 1.92 (07-064, assigned to *Paenibacillus fonticola*) to 6.09 (06-076, assigned to *Bacillus licheniformis*) (Table 3). Most of these cellulose-degrading strains were assigned to the genus *Bacillus* and were isolated from BSFL reared on EFB (30 strains), followed by PP (nine strains) and CPC (six strains). In total, 83.9% of all *Bacillus* strains were tested positive by Congo Red assay. Among the isolated *Bacillus* strains, strain 06-012 (assigned to *Bacillus subtilis*) had the lowest cellulolytic activity, whereas strain 06-076 (assigned to *Bacillus licheniformis*) had the highest activity. Several studies have confirmed the ability of *Bacillus licheniformis* to degrade cellulose (47–49). Therefore, we used strain 06-076 as an additional positive control. We also observed partial clearance of the cellulose substrate by strains 07-065 and 07-077 assigned to *Clostridioides mangenotii*. Despite being predicted to have the gene for endoglucanase, it was not proven *in vitro* that several isolates assigned to the family *Enterobacteriaceae* and *Rhizobiaceae* were able to degrade cellulose like the *Bacillaceae*.

**TABLE 3:** Identification of cellulose-degrading bacteria based on Congo Red assay^*a*^

Strain no.	Top BLAST hit	Similarity (%)	CMC degradation-Congo Red	Enzymatic activity index (EAI)
01-149	*Bacillus velezensis*	100	+	2.64
01-174	*Bacillus subtilis*	99.8	+	2.29
06-004	*Bacillus subtilis*	100	+	2.76
06-005	*Bacillus pumilus*	100	+	3.48
06-007	*Bacillus altitudinis*	99.5	+	3.05
06-009	*Bacillus subtilis*	100	+	2.74
06-012	*Bacillus subtilis*	100	+	2.21
06-014	*Bacillus subtilis*	99.9	+	2.32
06-017	*Bacillus subtilis*	100	+	4.43
06-019	*Bacillus subtilis*	100	+	3.34
06-020	*Bacillus subtilis*	99.8	+	3.86
06-021	*Bacillus subtilis*	100	+	3.00
06-033	*Bacillus sonorensis*	99.9	+	3.84
06-034	*Bacillus licheniformis*	99.5	+	2.63
06-037	*Bacillus licheniformis*	99.9	+	4.25
06-038	*Bacillus licheniformis*	99.5	+	3.70
06-039	*Bacillus licheniformis*	99.8	+	4.57
06-040	*Bacillus pumilus*	99.5	+	3.85
06-041	*Bacillus licheniformis*	100	+	3.36
06-042	*Bacillus licheniformis*	99.4	+	3.96
06-043	*Bacillus subtilis*	100	+	2.54
06-044	*Bacillus subtilis*	100	+	4.47
06-055	*Bacillus licheniformis*	99.5	+	3.95
06-061	*Bacillus subtilis*	100	+	4.00
06-064	*Bacillus paralicheniformis*	98.5	+	3.06
06-069	*Bacillus subtilis*	99	+	2.86
06-070	*Bacillus licheniformis*	99.6	+	3.78
06-071	*Bacillus licheniformis*	99.6	+	2.67
06-073	*Bacillus licheniformis*	99.7	+	5.23
06-076	*Bacillus licheniformis*	100	+	6.09
06-077	*Bacillus licheniformis*	99.6	+	3.75
06-092	*Bacillus paralicheniformis*	99.6	+	6.00
06-102	*Rhizobium nepotum*	97.24	+	2.38
07-048	*Bacillus amyloliquefaciens*	100	+	2.74
07-049	*Bacillus subtilis*	98.6	+	3.28
07-050	*Bacillus subtilis*	100	+	3.50
07-051	*Bacillus amyloliquefaciens*	100	+	3.63
07-052	*Bacillus subtilis*	99.7	+	3.43
07-053	*Bacillus subtilis*	99.4	+	2.84
07-064	*Paenibacillus fonticola*	98	+	3.19
07-065	*Clostridioides mangenotii*	99.1	(+)	3.12
07-068	*Paenibacillus vini*	99.8	+	2.92
07-077	*Clostridioides mangenotii*	98	(+)	3.71
07-101	*Aquamicrobium terrae*	99.9	+	2.13
10-022	*Populibacterium corticicola*	94.25	+	1.92
10-033	*Bacillus cereus*	99.89	+	3.04
10-034	*Bacillus cereus*	99.64	+	3.67
10-035	*Bacillus cereus*	100	+	3.44
10-036	*Bacillus cereus*	100	+	2.89
10-043	*Bacillus cereus*	99.80	+	3.25
10-045	*Bacillus cereus*	100	+	3.78
10-046	*Bacillus cereus*	100	+	4.16
10-048	*Bacillus cereus*	99.9	+	2.91
10-050	*Bacillus toyonensis*	98.94	+	2.60
10-100	*Aureimonas altamirensis*	99.79	+	2.94
10-101	*Sphingobacterium mizutaii*	99.82	+	2.22

^*a*^ Cellulose degradation in CMC media evaluated by Congo Red staining was sorted as follows: +strong, (+) partial clearance.

## DISCUSSION

Research shows that BSFL can degrade a variety of side streams (3, 19–21, 30, 32). Here, we used three different plant-based side streams and analyzed their chemical composition. Our objective was to observe how these diets influenced not only the life-history traits but also the cultivable microbes in the larval gut. In addition, we further evaluated the cellulose-degrading capability of the microbial isolates using function prediction followed by *in vitro* screening.

### The influence of various diets on BSF development

We evaluated the growth performance of BSFL reared on three side streams (EFB, CPC, and PP) and one standard diet (CF). In previous studies, BSFL have been fed on food waste, agricultural byproducts, and manure resulting in varying performance during developmental stages depending on the nutritional value of the substrates (19, 50–52). We found that BSFL survival rates were consistently high, up to 100% on all substrates. The high protein content of CPC (26.2% DM) resulted in early growth and rapid development (43 d to reach the emergence of the adults), similar to the control group (47 d). However, the final larval weight was ~50% lower compared to the control group. The presence of the toxic polyphenolic compound gossypol in the CPC may slow down the developmental process, thus affecting the larval weight (32). Although we found a high protein content, we also found a high fiber content in CPC (45.7%). Apart from the antinutritive gossypol, the difficult to digest fibrous part of the substrate, might also contribute to the lower final larval weight. By contrast, larvae fed on EFB and PP required longer to reach the prepupal stage and gained less weight than CF larvae, especially those on the EFB diet (~169 d). EFB has a low protein content and is rich in lignocellulose, making it difficult to digest, but in a previous work by Klüber et al. (23) pre-treatment with the white rot fungus *Bjerkandera adusta* was shown to increase the final larval weight by ~25%, showing that fermentation can improve the digestibility as well as the weight gain of larvae. On the other hand, larval weight in the PP group in this study was slightly higher than EFB and CPC larvae, probably reflecting the nutritional value of the PP, which is most likely attributed to residual starch in the PP. Meanwhile, Serena and Knudsen (53) reported that potato pulp contains high amounts of starch, but not as high compared to the grain product (wheat). BSFL reared on agricultural waste, fruit, and/or vegetables achieved a lower weight up to 50% at the end of development when compared to nutritious diets, such as chicken feed (9, 54–56). A study by Manurung et al. (57) revealed that BSFL also can survive on and digest rice straw from the post-harvest season at various feeding rates. However, the final larval weight was lower compared to BSFL reared on other organic wastes that are rich in fat and protein. Rice straw by itself is not suitable as animal feed due to the high lignocellulose content (13.5% lignin, 27.8% hemicellulose, and 24.0% cellulose) and low protein content of 3%–6% (58). Another study of BSFL reared on seaweed also had a lower weight at the end of their development (59). A lower weight but high survival rate was reported for BSFL reared on apple pulp, whereas larvae fed on a nutritious diet (CF, Gainesville diet, chicken start mash) reached a larval weight of up to 303 mg and had a shorter life cycle, which is advantageous for industrial processes (23, 32, 60). These findings demonstrate that the nutrient composition, in addition to environmental factors such as the substrate’s moisture content and pH, affects the development of BSFL by influencing biomass accumulation (61). It is possible that the low protein and fat levels combined with the high fiber content of the feed had a negative effect on the larvae, extending the feeding period prior to the prepupal stage as indicated by the correlation analysis. The larvae accumulate fat during their growth and do not feed at later stages (L6–adult); therefore, larval energy reserves are critical, which also influence adult life-history traits, including longevity and fecundity (Table 1).

Plant tissues are rigid in structure due to the content of lignocellulose, which is composed of lignin, cellulose, and hemicellulose (62). The lack of nutrients such as fats, proteins, and vitamins causes herbivorous animals, including insects, to adapt (63). Although they have a small number of lignocellulose-degrading enzymes, mutualistic interactions with microbial symbionts are often necessary for the efficient degradation of such compounds (64, 65). For example, wood- and soil-feeding higher termites strongly rely on cellulolytic gut microbes to digest their food efficiently (25–27). It has also been shown for the woodwasp *Sirex noctilio* (Hymenoptera: Siricidae) that bacterial symbionts may play an important role in the degradation of woody substrates and thus contribute to nutrient acquisition (66). Furthermore, Shil et al. (67) have reported the cellulolytic activity of 15 bacterial isolates obtained from the gut of the phytophagous grasshoppers *Oxya velox* (Orthoptera: Acrididae), *Aularches miliaris* (Orthoptera: Pyrgomorphidae), and the ladybug *Propylea quatuordecimpunctata* (Coleoptera: Coccinellidae). Larvae of the yellow mealworm, *Tenebrio molitor* (Coleoptera: Tenebrionidae), possess enzymes, including those derived from the gut microbiome, which enable the digestion of cell walls. This enables *T. molitor* to digest ~46% of the lignocellulosic components of straw (68). In *Coptotermes formosanus* (Blattodea: Rhinotermitidae), endogenous enzymes appear to collaborate with symbiotic protists to break down lignocellulose biomass (69). In *Odontotaenius disjunctus* (Coleoptera: Passalidae)*,* lignocellulose degradation takes place mostly in the midgut and hindgut, and the depolymerized products are then fermented in the anterior hindgut (70). In BSFL, the gut microbiome not only influences their ability to adjust to their diet but also plays a role in growth and development (3, 7). The gut microbiome in BSFL differs significantly in composition between groups fed on calf forage, cooked rice, and food waste (19). It has been demonstrated that inoculation with bacteria significantly promotes growth and development in germ-free BSFL lines (71). The ability of insect-associated microbes to sustain the growth and development of their hosts has also been confirmed in gnotobiotic house flies (*Musca domestica*; Diptera: Muscidae) (72, 73). Thus, we conclude that the gut microbiome plays a critical role in BSFL digestion of different side streams which vary in terms of nutritional value.

### Bacterial isolates from guts of BSFL reared on different diets

A cultivation-independent approach based on marker genes such as the 16S ribosomal RNA (rRNA) gene provides a general broad overview of microbial communities. However, to identify viable, metabolically active gut bacteria, we used a comprehensive cultivation-dependent approach. Previous studies have found a large proportion of culturable bacteria in BSFL reared on CF, using a variety of selective and non-selective media as well as aerobic and anaerobic cultivation techniques (9). Therefore, we followed the same cultivation procedure to analyze the culturable microbiome of BSFL reared on CPC, EFB, and PP and compared it with the collection of bacterial isolates obtained from larvae reared on CF.

Many of the taxa we isolated, including several species of *Aquamicrobium*, *Brevundimonas*, *Devosia*, *Clostridioides*, *Lacrimispora,* and *Fusicatenibacter*, have not been isolated from BSFL before. Whereas *Achromobacter* and *Bordetella* were isolated in previous studies (9, 74). *Clostridioides*, *Lacrimispora,* and *Fusicatenibacter* are obligate anaerobic taxa, which might be underrepresented in this cultivation-dependent study because their cultivation is more fastidious, and their growth is slower compared to most of the aerobic and facultative anaerobic strains in this strain collection. However, we obtained five isolates of obligately anaerobic *Lachnospiraceae* from BSFL reared on the CPC diet. *Lachnospiraceae* have also been detected by amplicon sequencing (relative abundance of 0.2%–2.4%, mean 1.0%) in BSFL reared on CPC in a previous study (32).

The strain collection reflects diet-dependent microbial shifts especially in BSFL reared on the high-fiber EFB diet, which featured a greater abundance of *Bacillus* strains compared to isolates from larvae reared on the nutrient-rich CF diet. Also, cultivation-independent studies of BSFL reared on CF revealed a generally low relative abundance (0.2%–1.3%, mean 0.7%) of the genus *Bacillus* (9) or even its absence (32). A high relative abundance of *Bacillus* (5.8%) has been observed in the midgut of *Trypoxylus dichotomus* (Coleoptera: Scarabaeidae) that feed on bamboo fiber (75). In addition, a similar trend was observed in *Protaetia brevitarsis* (Coleoptera: Scarabaeidae) which feeds on corn straw. The relative abundance of *Bacillaceae* was high in the midgut (50.4%) and hindgut (33.2%) (76).

### Fibrolytic potential of gut bacteria

PICRUSt2 results revealed that genes for endoglucanases are highly abundant in the reference genomes of the family *Bacillaceae* which also is the most abundant family of isolated bacteria from EFB rearing (Fig. 2 and 3).

Remarkably, 67.3% of all isolates from BSFL reared on the EFB diet were predicted to be capable of degrading cellulose and 48.4% of these (35% of all isolates) had cellulolytic activity confirmed *in vitro* by the Congo Red assay. By contrast, only 1% of the isolates from BSFL reared on CF were positive in the Congo Red assay. Moreover, in correlation to the crude fiber analysis, EFB has the highest fiber content resulting in the most isolates that confirmed to degrade cellulose *in vitro*, whereas CF results in both lowest fiber content and number of cellulose-degrading bacteria.

We also found that within the family *Enterobacteriaceae*, none of the isolates were positive for cellulose degradation *in vitro* although in many cases genes for endoglucanases were predicted, indicating that these bacteria have cellulolytic potential but are not necessarily using cellulose degradation pathways under *in vitro* conditions. This highlights the importance of pure cultures for the determination of bacterial functions in the gut and the studies that solely used the function prediction should be carefully interpreted.

Besides cellulose degradation, the culture collection obtained in this study could also be screened for further beneficial functions, for example, the synthesis of bioactive natural products and potential industrial applications such as the development of probiotics for insect farming.

No prediction for laccase was found using PICRUSt2. This supports the hypothesis that lignin degradation is mostly attributed to fungi and that BSFL gut bacteria may not be able to fully break down substrates with a high lignocellulose content like the EFB diet. This may also explain the slow development and low final weight of the BSFL reared on EFB compared to those reared on high-protein diets like CF and CPC. Furthermore, we found no prediction for exoglucanases, in agreement with previous studies (23). It might be possible to improve the growth performance of BSFL reared on EFB by fungal fermentation (23).

Thus far, little is known about the ability of black soldier fly-associated fungi to degrade lignocelluloses. Future studies should therefore focus more strongly on this part of the microbial community. By identifying fungal isolates, especially filamentous ones, their natural role in the BSFL digestion could be clarified and interesting candidates for biotechnological applications could be identified. In contrast to a fermentative pretreatment with white rot fungi, endogenous representatives could be investigated, which can also establish themselves in the intestinal microbiome of BSFL.

### Conclusion

Industrial side streams such as cottonseed press cake, empty fruit bunches, and potato pulp are often inefficiently used due to their low nutritional value. We have analyzed the chemical composition of these side streams and demonstrated that BSFL can be reared on them. Our findings revealed that the combination of high fiber content with low protein and fat levels negatively impacted the whole life stages of the black soldier fly, especially in the larval stage where the high fiber content extended the feeding period. Conversely, the side stream with higher protein levels significantly enhanced larval performance, as shown by the increased final larval weight.

Furthermore, the nutritional value of these side streams also affected the microbial communities within the larval gut. We have carried out the cultivation-dependent approach to assess the viable microbes and their cellulose-degradation capabilities. From BSFL reared on EFB, we isolated most strains that can break down cellulose, which correlates with the higher fiber content in EFB. This comprehensive approach allowed us to understand not only the impact of the side streams on the black soldier fly’s life-history traits but also the potential of their gut microbes in contributing to biodegradation processes, particularly cellulose degradation.

## MATERIALS AND METHODS

### Rearing BSF

BSF were provided by Bio.S Biogas (Grimma, Germany) in July 2018. Since then, the strain has been genetically isolated from other populations (>37 generations). Adult BSF were kept in 60 × 60 × 90 cm (l × w × h) mesh cages (Bioform, Nuremberg, Germany) located in the greenhouse at 26°C ± 1°C, 60% ± 5% relative humidity, and a 12-h photoperiod. To prevent the flies from drowning, water was provided *ad libitum* using water-soaked paper towels. Furthermore, the mesh cages were sprayed daily with water. Fresh egg clutches were harvested from artificial oviposition sites consisting of three wooden boards spaced by washers and held with rubber bands (77). The eggs were weighed using an ALJ 160–4A balance (Kern & Sohn, Balingen, Germany), placed in 19.5 × 16.5 × 9.5 cm plastic boxes (150 mg eggs = ~6,000 eggs per box), and sprayed with water. Once ≥50% of the eggs had hatched, larvae were fed initially with 10 g ground feed. Boxes were checked daily for remaining water and feed, and additional feed was provided *ad libitum* (usually at 48-h intervals) until the population reached the prepupal stage. The moisture content of the diets was measured using a TMT-MC-7828S soil moisture meter (OCS.tec, Neuching, Germany) and adjusted to ~70% by spraying. The larvae were reared in a climate chamber at 27°C ± 1°C and 65% ± 5% relative humidity in constant darkness (23). To prevent contamination, disposable nitrile gloves were worn throughout the experiment to handle and administer the feed.

### Experimental diets and influence on life history traits

Four diet groups were established: CF, CPC, EFB, and PP. CF was used as the control diet (GoldDott Eierglück, DERBY Spezialfutter, Muenster, Germany) because it is frequently described as a high-quality complete feed for the rearing of BSFL (78, 79). CPC—a side stream arising from the mechanical extraction of *Gossypium hirsutum* seeds—was purchased from a cotton ginning and oil mill factory (Kafantaris-Papakostas, Karditsa, Greece). Before shipping, it was stored in a dry warehouse for a few days up to a few months. EFB—a side stream of the palm oil industry created by threshing the fresh fruit bunches—was provided by PT Alternative Protein Indonesia (Tebet, Indonesia). Before shipping, it was dried in the open air and coarsely chopped into 20–30 cm fragments. After delivery, dry CF, CPC, and EFB (moisture content 7%–9%) were stored at room temperature in separate airtight plastic barrels until further use. PP was provided by the Fraunhofer Institute for Process Engineering and Packaging IVV (Freising, Germany). Potatoes were peeled under flowing water using a Flott 16K potato-peeling machine (Flottwerk, Rotenburg a.d. Fulda, Germany), followed by juicing using an HR1921/20 centrifugal juicer (Philips, Amsterdam, Netherlands). The potato juice was then processed to extract starch and protein. PP is a byproduct after the juicing process and was used directly as BSFL feed. It was stored in vacuum-sealed plastic bags at –20°C before use.

Prior to feeding the larvae, the CF, CPC, and EFB diets were ground to a comparable particle size (100–1,500 µm). CF was homogenized in a Mockmill 200 grain mill (Wolfgang Mock, Otzberg, Germany), CPC in an EGK200 spice and coffee grinder (Rommelsbacher, Dinkelsbühl, Germany), and EFB in an SM 2000 cutting mill (Retsch, Haan, Germany). The particle size was determined using analytical sieves (Retsch). PP was already homogenized by juicing (moisture content ~80%). A portion of the PP was dried in a BDA-15dehydrator (Beeketal Lebensmitteltechnik, Rastdorf, Germany) at 50°C, ground using the spice and coffee grinder, and added to the feed to adjust the total moisture content to 70%–75% (optimal rearing conditions).

All life-history traits were recorded as biological triplicates for each diet group. Data from the CF and CPC groups (except for fecundity and longevity) have been published previously (23, 32). To determine the hatching time and record growth curves, we placed 150 mg of eggs in three replicate containers per diet. Growth measurements were started as soon as the larvae reached a manageable size of 3–4 mm (instar L3, 5–10 d), which strongly depends on the diet. The six larval instars and pupae were differentiated using standardized morphological characteristics (length, weight, head capsule diameter, coloring), as described elsewhere (80). The mean weight of 50 randomly selected larvae was determined at 48-h intervals using an AT261 DeltaRange analytical balance (Mettler, Giessen, Germany) until ≥50% of the population reached the prepupal stage. The survival rates of the stages larva–prepupa, prepupa–pupa, and pupa–imago were examined in 19.5 × 16.5 × 9.5 cm plastic boxes, each containing 100 individuals. The transition between two developmental stages was assumed as soon as ≥50% of the individuals reached the next developmental stage. Because prepupae and pupae do not feed, we provided feed only for the evaluation of larval development (L1–L5). We documented the final weight (as above) and final length (using VHX-2000 and VHX-4000 digital microscopes; Keyence, Osaka, Japan) of 50 L5 larvae, prepupae, pupae, and 60 imagines. Pupae that did not develop into flies within 1 month were considered dead. Within 24 h of emergence, adult flies were collected using spring steel tweezers, cold-inactivated by placing them on ice, and stored at –20°C until they were measured. In this context, the sexes of adult BSF were distinguished based on external dimorphisms, particularly the terminal genitalia and antennae (81).

The reproductive parameters were examined in 30 temporally synchronized (within 24 h after emergence) pairs of adult BSF kept under constant conditions in the greenhouse (26°C ± 1°C, 60% ± 5% relative humidity, 12-h photoperiod). Each pair was transferred in its own conical 8.4 × 8.4 × 11.4 cm plastic box, containing a 4 × 4 cm wooden board that served as a simplified oviposition site. If one of the individuals in a pair died within the first day after the experiment had started, they were discarded and replaced with a new pair. The lid was fitted with a circular 4.5 cm mesh insert, enabling gas exchange and the daily provision of water by spraying (~2 mL), as previously described (82). When spraying, the sides of the box were meticulously targeted to avoid harming the flies with the spraying pressure. The mating behavior of BSF is light-dependent, which is why all boxes were placed directly under a SON-K 400 high-pressure sodium-vapor lamp (DH Licht, Wülfrath, Germany) that illuminated the flies between 8:00 a.m. and 8:00 p.m. The lamp had the following technical specifications: 465 W power, 56,500 lm luminous flux, 725 µmol∙s^−1^ photon flux, and 2,000 K light color temperature. The light intensity was set to 300 µmol·m^−2^·s^−1^. Oviposition and sex-specific lifespan were checked daily. Females laying eggs were not disturbed. Freshly deposited egg clutches were collected, declustered carefully using metal spatulas, counted under an S9i stereomicroscope (Leica Microsystems, Wetzlar, Germany), and weighed.

### Chemical composition of feed substrates and BSFL

All parameters were recorded as triplicates. For nutritional analysis, L5 larvae (~100 g) and feed substrates (~20 g) were ground using a mortar under liquid nitrogen and lyophilized for 72 h. Before this, the moisture content of the homogenized samples was determined thermogravimetrically using an M35 Moisture Analyzer (Sartorius, Göttingen, Germany). The following parameters relate to the dry matter. We used 0.5 g of lyophilized larvae and 1 g of lyophilized feed to analyze the levels of crude ash, protein, and fat. For crude fiber determination, 3 g of lyophilized feed was used.

For crude ash determination, samples were pre-incinerated in a quartz crucible over a Bunsen burner, incinerated twice for 6 h at 550°C in an L 9/11 muffle furnace (Nabertherm, Lilienthal, Germany), and the content was calculated using differential weighing. Total nitrogen analysis was conducted according to the Kjeldahl method (83). For this purpose, the samples were digested with sulfuric acid (Kjeldatherm, Gerhardt, Königswinter, Germany), followed by automated steam distillation and titration using the Vapodest 500 (Gerhardt, Königswinter, Germany) and TitroLine 5000 (SI Analytics, Mainz, Germany) systems. The crude protein content was calculated based on a conversion factor of 6.25, as suggested elsewhere (84). For crude fat determination, samples were disintegrated manually for 30 min in 150 mL boiling 4 mol⋅L^–1^ HCl, filtered, washed to neutralize using hot demineralized water, and dried for 2 h at 105°C. The fat was then extracted automatically with *n*-hexane in a Soxtherm system (Gerhardt, Königswinter, Germany) and the content was determined gravimetrically. The crude fiber content was determined according to the method of Scharrer-Kürschner, as described elsewhere (85).

### Cultivation, isolation, and identification of gut bacteria

BSFL in the weight range of 100–150 mg (L5) were washed with 70% ethanol and sterile water, and the guts were dissected with sterile forceps under a stereomicroscope. Five guts were pooled, homogenized, and serially diluted. We used the same cultivation media and protocols as reported elsewhere (9). Cultures were preserved by preparing stocks in Roti-Store cryo-vials (Carl Roth, Karlsruhe, Germany) or glycerol (50%) followed by storage at –80°C.

DNA was extracted from pure cultures in 0.2% sodium dodecylsulfate (SDS) at 95°C for 10 min and diluting 1:10 in nuclease-free water. For Gram-positive species, cells were disrupted by bead beating in a FastPrep-24 device (MP Biomedicals, Solon, OH, USA) for 90 s at 6.5 m∙s^−1^, followed by extraction using the NucleoSpin soil kit (Macherey-Nagel, Düren, Germany), according to the manufacturer’s manual. The 16S rRNA genes were amplified using the bacteria-specific 27F (5′-AGA GTT TGA TCM TGG CTC AG-3′) and 1492R (5′-ACG GYT ACC TTG TTA CGA CTT-3′) primers and were sequenced using the Sanger method as previously described (9).

Sequences were evaluated and trimmed using Geneious v10.2.6 (Biomatters, Auckland, New Zealand). Some sequences lacked either a forward or backward read, thereby forward and/or backward reads were used for taxonomic identification. Read 06-070_27f was removed (<150 bp) and only the sufficient backward read with 1,135 bp was used. Five pairs of identical forward sequences with no available backward reads to differentiate were handled as one isolate. The sequences from CF rearing (9) were used and merged as provided by the NCBI database. All sequences were identified using a BLAST search of the NCBI nucleotide database (http://www.ncbi.nlm.nih.gov/blast) in Geneious (https://www.geneious.com) with default settings. The individual results were then manually combined to achieve the best possible identification of the isolated bacterial strains (Table S1). Isolate 01-177 (assigned to *Neglecta* sp. Marseille-P3890) and 06-054 (assigned to *Enterobacter pulveris*) were manually renamed to *Scatolibacter rhodanostii* and *Franconibacter pulveris* (86, 87). Furthermore, one isolate (01-040) from a previous study (9) was assigned to the genus *Wohlfahrtiimonas* (99.9% similarity). The closest related strain is *Koukoulia aurantiaca*; however, it is yet to be described (88).

### Prediction of microbial functions

Forward, backward and the provided merged sequences from CF rearing were used for functional predictions. All four groups were analyzed using the standalone version of PICRUSt2 v2.5.2 (89). We defined all unique sequences as operational taxonomic units (OTUs) with an abundance of 1 and used the PICRUSt2 numerical values solely as an indicator of the form present or not if zero. We filtered the results according to the EC or KO identifiers we defined for the presence of endoglucanases, suggesting the ability to break down cellulose (Table S1). The individual results were then manually combined for the best possible result.

### Screening of cellulose-degrading strains from the BSFL gut

Microbial strains isolated from the BSFL gut were inoculated in duplicates on carboxymethyl cellulose (CMC) agar (modified ATCC medium 2720) with the following composition: 0.5 g (NH_4_)_2_SO_4_, 0.5 g l-asparagine, 1 g KH_2_PO_4_, 0.5 g KCl, 0.2 g MgSO_4_, 0.1 g CaCl_2_, 0.5 g yeast extract, 20 g agar and 10 g CMC in 1 L distilled water, pH 6.5. *Cellulomonas flavigena* (DSM20109) and *Bacillus licheniformis* (06-076) were used as positive controls. The plates were incubated at 27°C for 48 h before measuring the diameter of colonies and imaging. The plates were then flooded with 0.1% Congo Red and incubated for 30 min at room temperature. After removing the staining solution, the plates were washed with 1 M NaCl and incubated for 2 × 10 min at room temperature. The formation of a clear zone indicated the degradation of CMC (90).

All strains tested positive (showing a clear zone) were confirmed by a repeating trial (Fig. S1) and the enzymatic activity index (EAI) was determined according to Anagnostakis and Hankin (91). The sum of the colony diameter and the clear zone diameter was divided by the diameter of the colony to calculate the enzymatic activity index (EAI). Strains with an EAI > 2.5 are considered producers of cellulolytic enzymes (92).

### Data analysis and statistics

Data were visualized and statistical significance was calculated using Excel 2016 (Microsoft, Redmond, WA, USA), OriginPro 2023 (OriginLab, Northampton, MA, USA), and R v4.2.2 with package tidyverse v1.3.2 (93). Lifetime-related parameters (including sex-specific and total adult longevity) were assessed using the non-parametric Kaplan–Meier estimator. The *S(t*) survival functions were compared pairwise using log-rank tests (α = 0.05). The homogeneity of variance was evaluated using Levene’s test. The remaining temporal, physiological, and reproductive parameters, as well as differences in the chemical composition of the diets and larvae, were subjected to a one-way analysis of variance (ANOVA). Here, means were separated using the Bonferroni–Holm test (homogeneous variance). If the homogeneity of variance assumption was not met, we applied Welch’s one-way ANOVA followed by a Games-Howell *post hoc* test (94). Linear relationships were calculated using the Pearson product-moment correlation (95).

## Data Availability

Raw sequencing data are publicly available under BioProject PRJNA1085536 at the NCBI database. Sequence 06-070_27f was not uploaded as it did not meet GenBank requirements (<150 bp). Sequences 10-041_1492r and 10-115_27f were trimmed of ambiguous bases during upload. The previously published data used (9) are also available at the NCBI database under the accession numbers MZ413999-MZ414160 as part of BioProject PRJNA739514.

## SUPPLEMENTAL MATERIAL

Data Set S1 aem.00991-24-s0001.xlsx Metadata of the gut isolates from BSFL.

Fig. S1 aem.00991-24-s0002.tif Congo Red assay repeating trial as positive confirmation of gut bacterial isolates from BSFL.

Fig. S2 aem.00991-24-s0003.tif Workflow of cultivation-dependent approach and Congo Red assay.

Supplemental legends aem.00991-24-s0004.docx Legends for Fig. S1 and Fig. S2.

ASM does not own the copyrights to Supplemental Material that may be linked to, or accessed through, an article. The authors have granted ASM a non-exclusive, world-wide license to publish the Supplemental Material files. Please contact the corresponding author directly for reuse.
