# Supplementary figures and images for: Turning trash into treasure: Hermetia illucens microbiome and biodegradation of industrial side streams
Source: Appl Environ Microbiol. 2024 Oct 22;90(11):e00991-24. doi: 10.1128/aem.00991-24 (PMC11577765; doi:10.1128/aem.00991-24)

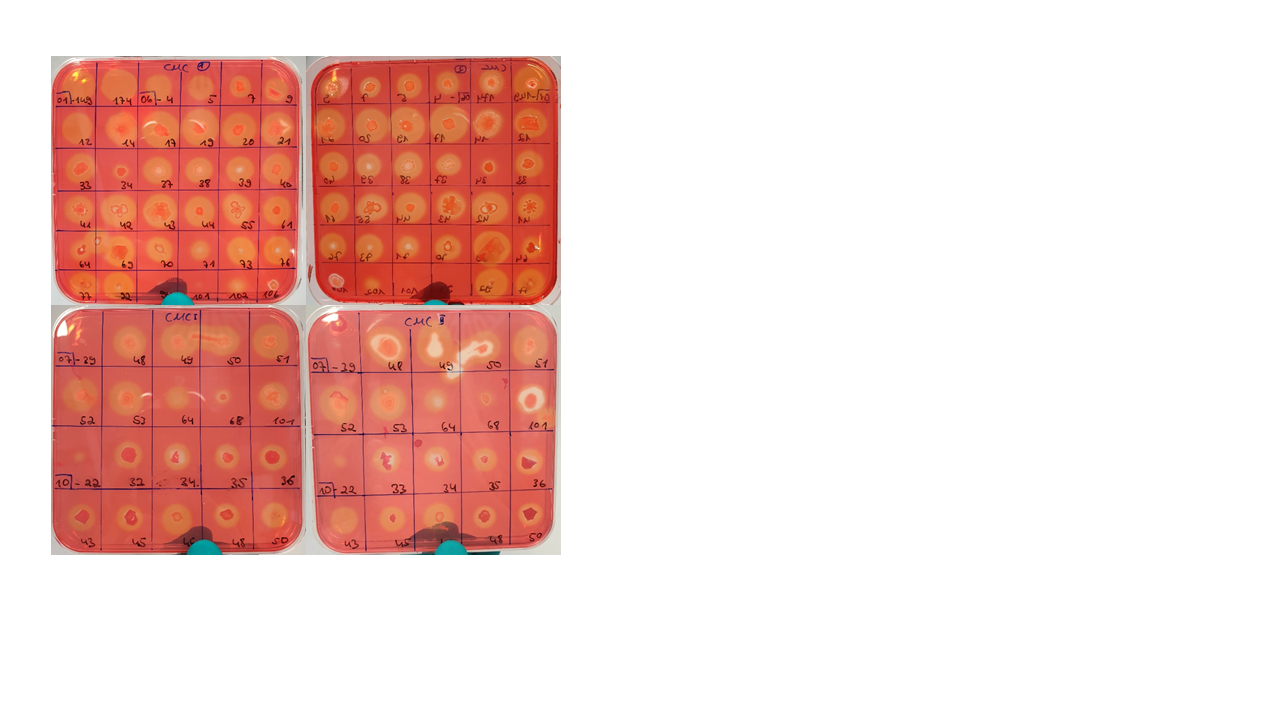

Supplement: Fig. S1 — Congo Red assay repeating trial as positive confirmation of gut bacterial isolates from BSFL. [file aem.00991-24-s0002.tif]

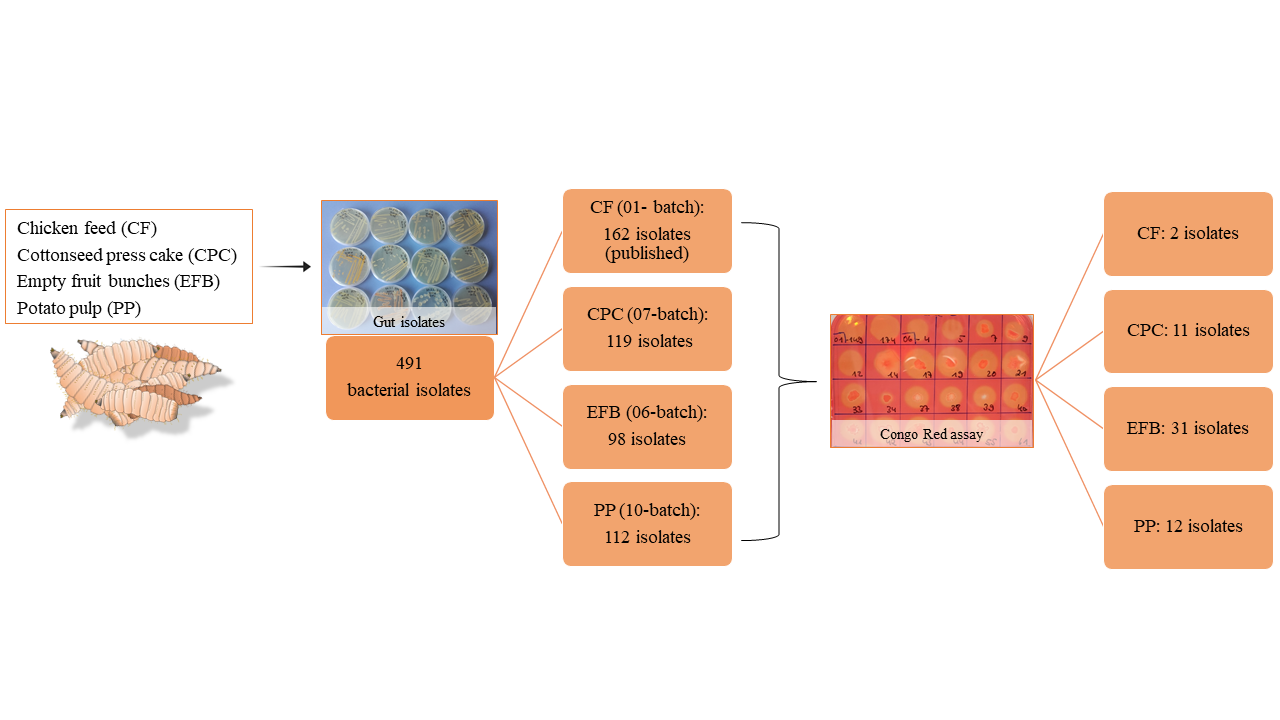

Supplement: Fig. S2 — Workflow of cultivation-dependent approach and Congo Red assay. [file aem.00991-24-s0003.tif]
