# Supplementary material for: Turning trash into treasure: Hermetia illucens microbiome and biodegradation of industrial side streams
Source: Appl Environ Microbiol. 2024 Oct 22;90(11):e00991-24. doi: 10.1128/aem.00991-24 (PMC11577765; doi:10.1128/aem.00991-24)
Supplement: Supplemental legends — Legends for Fig. S1 and Fig. S2. [file aem.00991-24-s0004.docx]

**
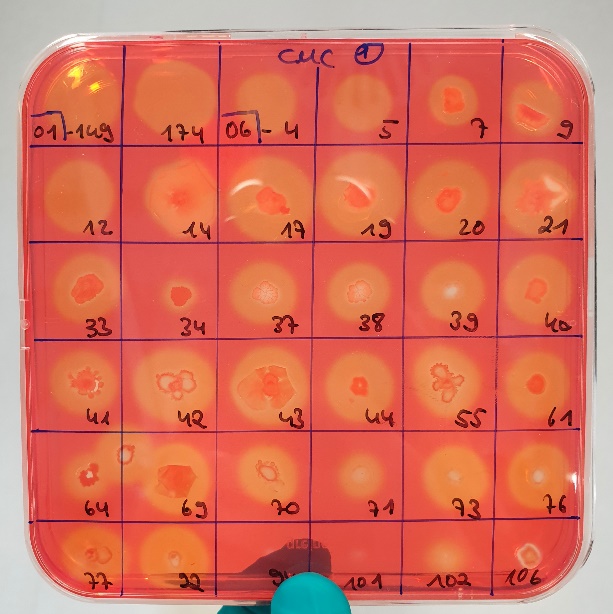

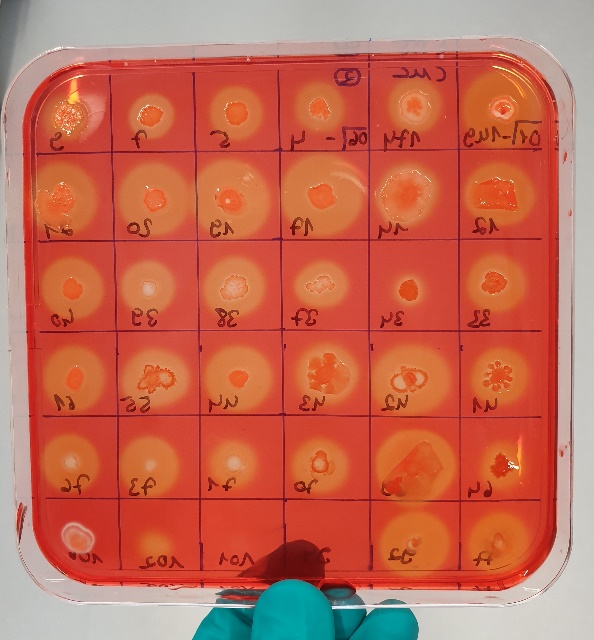
**


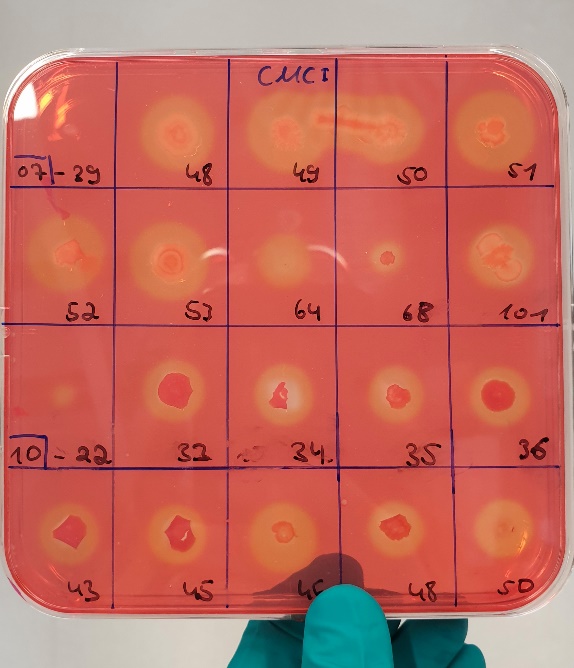
**
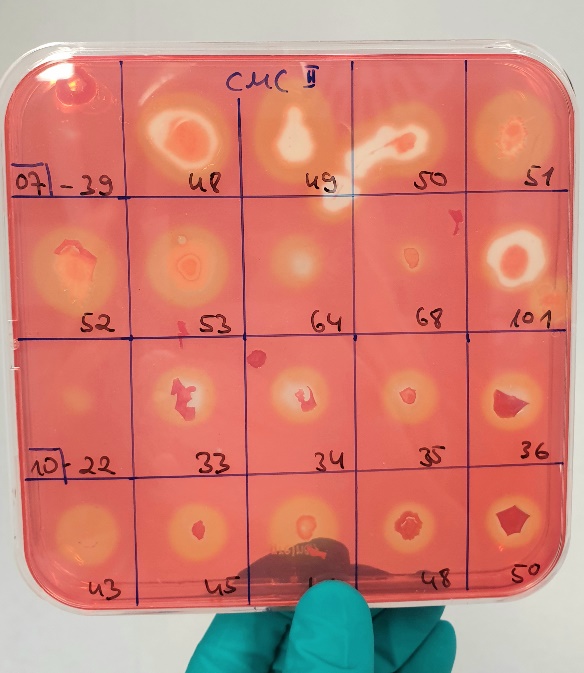
**

**FIGURE S1** Congo Red assay repeating trial as positive confirmation from CF (01-batch), EFB (06-batch), CPC (07-batch), and PP (10-batch) in duplicate (top and bottom left: 1^st^ replicate, image taken from bottom part of the square plate; top and bottom right: 2^nd^ replicate, image taken from inside of the square plate and image taken from bottom part of the square plate).


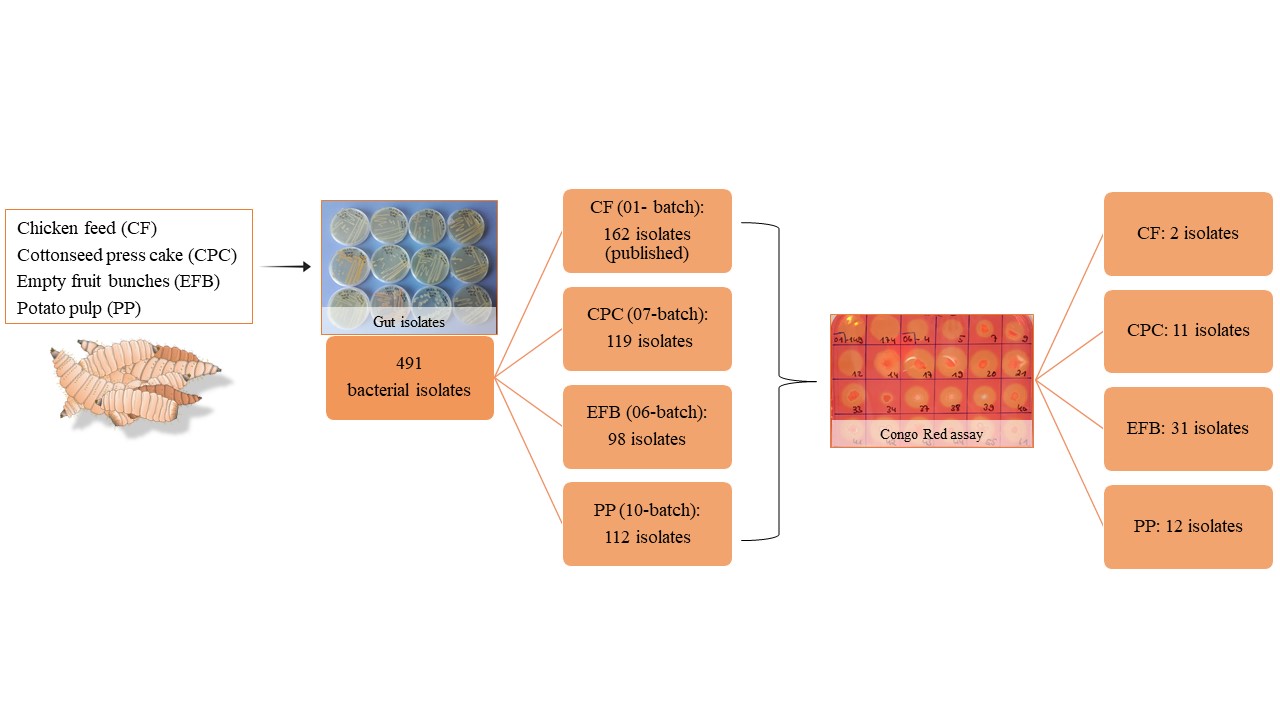


**FIGURE S2** Workflow of cultivation-dependent approach and Congo Red assay. From the left, the gut of BSFL from the respective diets was dissected, pooled, homogenized and serially diluted resulting in total of 491 bacterial isolates. Then, all gut isolates were screened for their cellulose-degradation ability using Congo Red assay, resulting in fewer number of isolates (right side).
